# The impact of multiple chronic diseases on ambulatory care use; a population based study in Ontario, Canada

**DOI:** 10.1186/1472-6963-12-452

**Published:** 2012-12-10

**Authors:** Elizabeth Muggah, Erin Graves, Carol Bennett, Douglas G Manuel

**Affiliations:** 1 C.T. Lamont Primary Health Care Research Centre, Élisabeth Bruyère Research Institute, 43 Bruyère Street, Ottawa, ON K1N 5C8, Canada; 2 Department of Family Medicine and Epidemiology and Community Medicine, University of Ottawa, 725 Parkdale Ave, Ottawa, ON K1Y 4E9, Canada; 3 Ottawa Hospital Research Institute, 725 Parkdale Ave, Ottawa, ON, K1Y 4E9, Canada; 4 Institute for Clinical Evaluative Sciences, 725 Parkdale Ave, Ottawa, ON, K1Y 4E9, Canada; 5 Health Analysis Division, Statistics Canada, 725 Parkdale Ave, Ottawa, ON, K1Y 4E9, Canada; 6 Dalla Lana School of Public Health, University of Toronto, 725 Parkdale Ave, Ottawa, ON, K1Y 4E9, Canada

**Keywords:** Multiple chronic disease, Primary health care, Burden of care

## Abstract

**Background:**

The prevalence of multiple chronic diseases is increasing and is a common problem for primary health care providers. This study sought to determine the patient and health system burden of multiple chronic diseases among adults in Ontario, Canada, with a focus on the ambulatory health care system (outpatient primary health care and specialist services).

**Methods:**

This population-based study used linked health administrative data from Ontario, Canada. Individuals, aged 20 years or older, who had a valid health card, were included. Validated case definitions were used to identify persons with at least one of the following nine chronic diseases: diabetes, congestive heart failure, acute myocardial infarction, stroke, hypertension, asthma, chronic obstructive lung disease, peripheral vascular disease and end stage renal failure. Prevalence estimates for chronic diseases were calculated for April 1, 2009. Ambulatory physician billing records for the two-year period, April 1, 2008 to March 31, 2010, were used to identify the number of outpatient ambulatory care visits.

**Results:**

In 2009, 26.3% of Ontarians had one chronic disease, 10.3% had two diseases, and 5.6% had three or more diseases. Annual mean primary health care use increased significantly with each additional chronic disease. Overall, there were twice as many patient visits to primary health care providers compared to specialists across all chronic disease counts. Among those with multiple diseases, primary health care visits increased with advancing age, while specialist care dropped off. While persons with three or more diseases accounted for a disproportionate share of primary health care visits, the largest number of visits were made by those with no or one chronic disease.

**Conclusions:**

The burden of care for persons with multiple chronic diseases is considerable and falls largely on the primary health care provider. However persons with no or one chronic disease are responsible for the largest number of ambulatory health care visits overall. Continued investment in primary health care is needed both to care for those with multiple diseases and to prevent the accumulation of chronic diseases with aging.

## Background

Chronic diseases, such as diabetes, heart disease and asthma, are the most prominent health care issues affecting Canadians [1]. There is growing recognition that chronic diseases do not occur in isolation, due, in large part, to shared risk factors and the accumulation of diseases with age [2-4]. In Canada, among people with chronic diseases, 49% of adults aged 65–79 years and 59% of adults aged 80 years and older report having at least two select diseases [4]. While patients with multiple diseases have their acute care needs met in the hospital, the majority of their long-term health care needs are managed by the primary health care providers [5]. Primary health care plays a central role in the treatment of chronic disease and also the prevention of disease and disease complications. Evidence indicates that timely and effective primary health care can improve health outcomes for persons with chronic diseases including fewer expensive hospitalizations and emergency department visits [6,7]. A better understanding of the impact of multiple chronic diseases on the primary health care system has been identified as a priority by decision makers and researchers [3,8,9].

To date, research exploring the burden of disease on the primary health care system in Canada [3-5] has been largely based on data from population health surveys which are subject to recall bias and sampling errors and do not always distinguish between outpatient visits to primary health care and specialist physicians. The use of health administrative data allows for a unique view of actual patterns of health care use at a population level. Typically research with health administrative data categorizes people by level of overall disease morbidity and does not explore outcomes by disease count, in part because it can be a challenge to accurately ascertain diseases using administrative data. Starfield et al. used administrative data to explore outpatient health care utilization in the United States and found a striking increase in primary health care use among younger (<65 years), but not older adults (>65 years), with increasing levels of morbidity [10]. Broemeling et al. used administrative data to explore the health care utilization by persons in British Columbia [11]. In that study, persons with a high burden of comorbid chronic diseases had more than three times the number of physician visits compared to the population average.

We sought to extend what is known about the burden of multiple chronic diseases by using highly validated disease ascertainment methods to determine the association of chronic disease count and patterns of ambulatory care use (primary health care and specialist) for the population of Ontario, Canada.

## Methods

This study was conducted using population-based data from the province of Ontario, Canada with a current total population of more than 13 million people [12]. Data were obtained from the linkage of several administrative databases, including hospital discharge abstracts and physician claims for ambulatory visits (i.e. outpatient data), housed at the Institute for Clinical Evaluative Sciences (ICES). The ICES databases are individually linked using an anonymous identification number in accordance with the provincial Personal Health Information Protection Act.

This study was approved by The Ottawa Hospital Research Ethics Board.

### Population

We included all living adults, aged 20 years or older, identified in the Ontario Registered Persons Database who had a valid Ontario health card on April 1, 2009.

To minimize inclusion of people who had potentially moved out of province, we excluded individuals who made no contact with the Ontario health care system within the five years prior to 2009. This criterion was evaluated by applying the five-year date of last contact restriction to all Ontario respondents from three cycles of the Canadian Community Health Survey (conducted in 2001, 2003 and 2005). Of more than 150,000 respondents of the Canadian Community Health Survey, 99.8% of individuals who answered the survey had contact with the health care system within the five years prior to writing the survey. While this approach may exclude certain groups that have less contact with the health care system, such as younger, less sick adults, we feel that it ensures the most accurate estimate of the population residing in Ontario.

**Figure 1:**
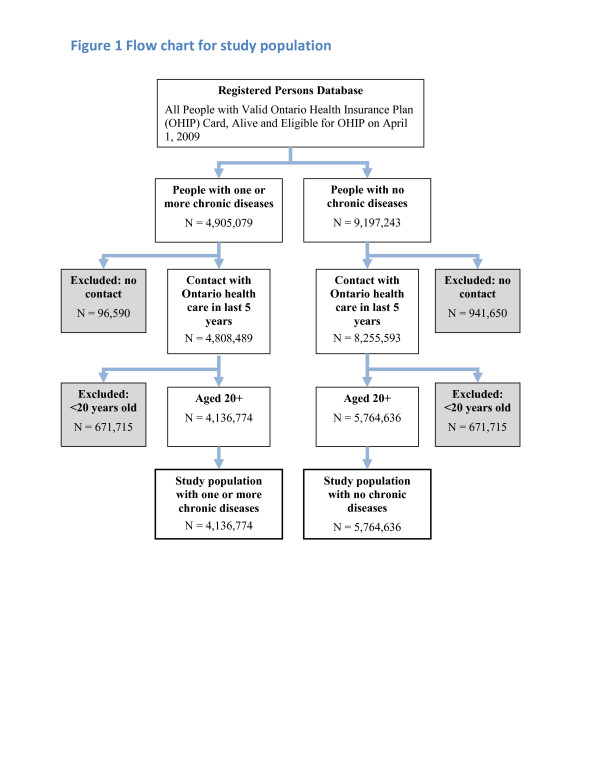
Flow chart for study population.

Figure 1 provides details of the sampling method.

### Chronic disease ascertainment

To identify persons in our population with a chronic disease we used pre-existing, validated health administrative data case definitions for the following nine chronic diseases: diabetes, congestive heart failure, acute myocardial infarction, stroke, hypertension, asthma, chronic obstructive lung disease, peripheral vascular disease and end stage renal failure. The specific details on the methods of disease ascertainment, including the technical case definitions used, are presented elsewhere [13-19]. Prevalence estimates for the number of chronic diseases were calculated for April 1, 2009. All adults in the Registered Persons Database who were eligible for provincial health insurance and had contact with the health care system in the preceding five years served as the denominator.

### Health care utilization

We used provincial billing records to identify the number of outpatient health care visits made to primary care and specialist physicians within a two-year period (April 1, 2008 to March 31, 2010) and presented results as an annual rate. Due to the high number of outpatient visits, the analysis was completed on a 25% random sample of the total study population.

### Data analysis

We calculated chronic disease prevalence by disease count in the study population. To explore the health care burden on the patient, we calculated the mean annual number of ambulatory health care visits (primary health care and specialist) per person and stratified results by disease count. The total annual number of ambulatory health care visits for all persons in each disease count group was calculated to provide an estimate of the overall burden on the primary health care system. The impact of age on utilization was estimated by stratification of results across age categories. All analysis was done in SAS version 9.2 (SAS Institute, Cary, NC).

## Results

**Figure 2:**
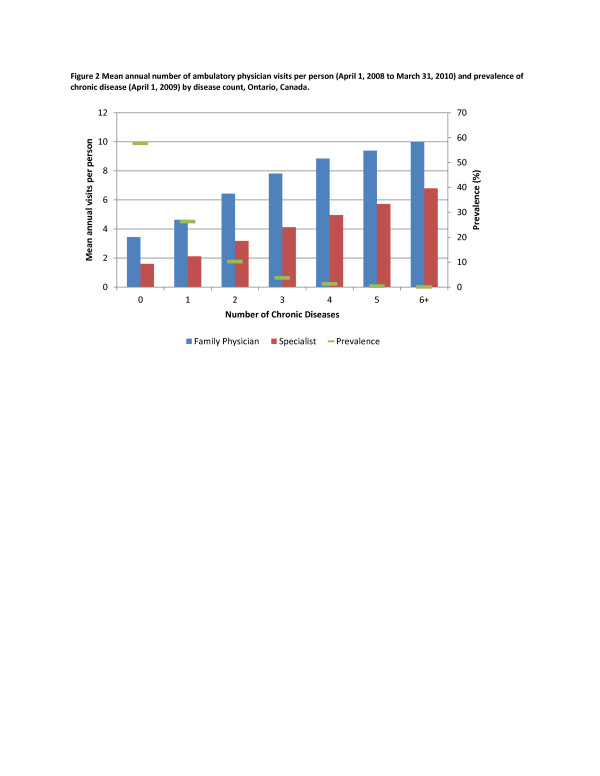
Mean annual number of ambulatory physician visits per person (April 1, 2008 to March 31, 2010) stratified by age, Ontario, Canada.

The prevalence of chronic diseases by disease count in Ontario on April 1, 2009 is presented in Table 1. Just over 26% of Ontarians had one chronic disease, 10.3% had two diseases and 5.6% had three or more diseases. Table 1 also presents the total number of primary health care visits to primary care physicians and specialists by disease count over the study period. There were twice as many primary health care visits, compared to specialist visits, across all disease counts. While persons with three or more diseases accounted for a disproportionate share of ambulatory visits (both primary health care and specialist), the largest absolute number of visits was made by those with no or one chronic disease (Figure 2).

**Table 1:** Prevalence of chronic disease by disease count (April 1, 2009) and number of ambulatory health care visits for Primary Care and Specialist physicians made between April 1, 2008 and March 31, 2010 (N=28,450,000)

**Number of chronic diseases**	**Prevalence**	**Primary health care**		**Specialist**	
		Number of visits*	**% of all ambulatory visits**	Number of visits*	**% of all ambulatory visits**
**0**	57.8%	7,830,000	27.6%	3,640,000	12.8%
**1**	26.3%	5,940,000	20.9%	2,750,000	9.7%
**2**	10.3%	3,240,000	11.4%	1,600,000	5.6%
**3**	3.7%	1,390,000	4.9%	737,000	2.6%
**4**	1.3%	568,000	2.0%	315,000	1.1%
**5**	0.4%	199,000	0.7%	117,000	0.4%
**6+**	0.2%	73,100	0.3%	51,000	0.2%

*Analysis based on a 25% random sample of the total study population.

**Figure 3:**
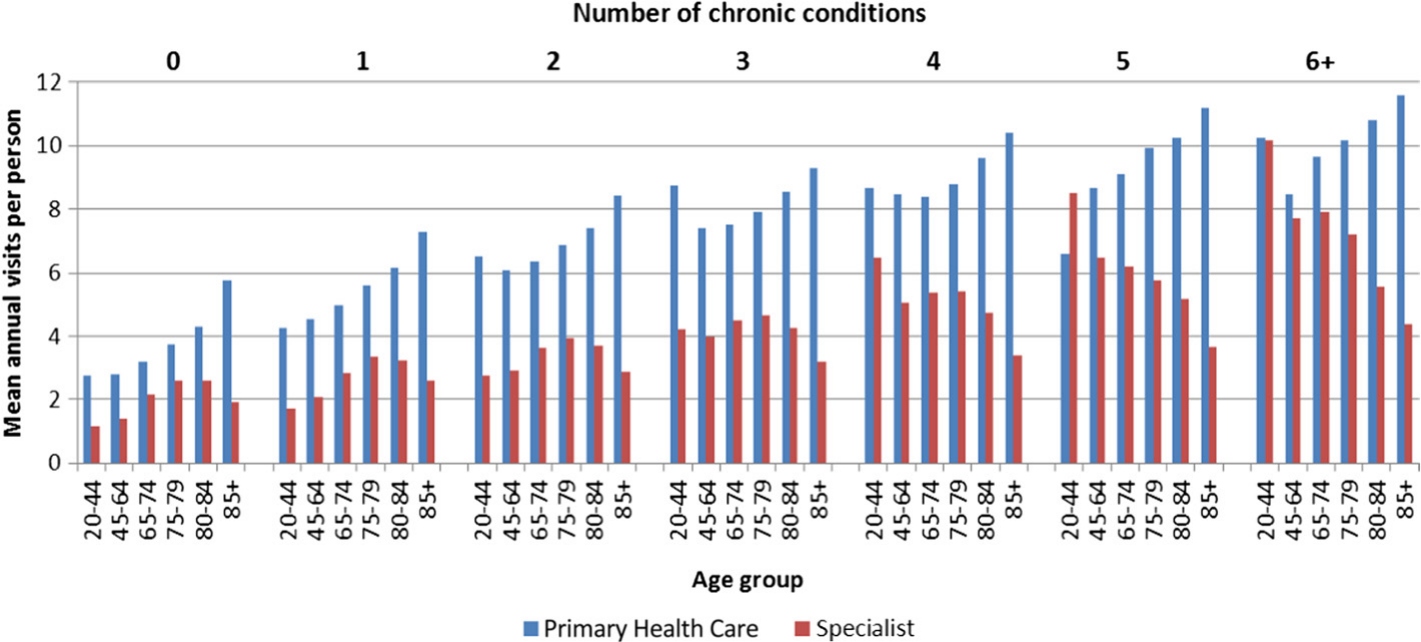
Mean annual number of ambulatory physician visits per person (April 1, 2008 to March 31, 2010) and prevalence of chronic disease (April 1, 2009) by disease count, Ontario, Canada.

There is an increase in the mean number of health care visits to primary health care physicians with age, while health care visits to specialist physicians drop off at the highest age categories (Figure 3). For example, young adults (aged 20–44 years) with 6 or more diseases had 10 primary health care and 10 specialist visits per year, compared to those aged 85 years and older with 6 or more diseases, who had 11 primary health care visits and only 4 specialist visits per year.

## Discussion

The objective of this study was to describe the burden of multiple chronic diseases on the patient and the ambulatory health care system. Our results demonstrate three key trends. First, while the burden of disease on the patient, as measured by mean number of ambulatory health care visits per year, was higher for those with multiple chronic diseases, the overall impact on the primary health care system was relatively small. Second, across all disease counts and ages there were many more visits to primary health care physicians compared to specialist physicians. This trend was evident even among the most elderly patients who, with age, had ever-increasing visits to primary health care physicians, while the number of visits to specialist physicians declined. Third, we found that, while the number of primary health care visits increased with age, the elderly with no diseases had many fewer visits per year, compared to younger adults with multiple chronic diseases.

Our findings confirm what other research has suggested about the high burden of chronic disease on the individual patient [3,4,11]. Recently, the Canadian Institute for Health Information reported results of a national survey that found more than twice the rate of reported visits to a family doctor among seniors with three or more diseases, compared to seniors with only one chronic disease [3]. Using data from the 2005 Canadian Community Health Survey, those with multiple chronic diseases represented 12% of the population but they accounted for 24% of all primary health care visits and 25% of all specialist consultations [4]. In that study the mean annual number of consultations with a family doctor was 4.0 in general population, 3.1 for those with no diseases, 4.7 with 1 disease and 7.4 for those with three or more diseases; estimates that are very close to our results.

Importantly, our study demonstrates that patients with multiple chronic diseases do not drive overall primary health care use in Ontario because prevalence of multiple chronic diseases in the population remains low. The majority of primary health care visits continue to be made by adults who have no or one disease. Future research could assess time trends to see if this pattern is changing, given the predicted rise in the prevalence of multiple chronic diseases with the aging population.

We acknowledge that a number of important chronic diseases were excluded from this study, such as depression and joint disease. This may have resulted in an overestimation of the number of persons with no or one disease. Although the exclusion of these diseases may change prevalence estimates and the associated number of visits, we do not believe this has an impact on the patterns of health care utilization we found. If anything, our results are an underestimation of the overall burden of multiple chronic diseases on the primary health care system. Validation studies for the ascertainment methods for additional chronic diseases in the Ontario databases are an ongoing effort at ICES.

Canada has a strong primary health care system and primary health care providers act as gatekeepers to specialist services. Thus, it was not surprising to see that there were more primary health care visits in our study, particularly among the very elderly. The lower rate of specialist visits in the very elderly can be explained by factors such as fewer new disease diagnoses that require specialist consultation and primary care provider or patient preference for less intervention and testing. Our results are different from a U.S. study of Medicare beneficiaries where the burden of care for non-elderly patients (<65 years) with high levels of morbidity fell disproportionately on primary health care but this pattern did not hold for the elderly who had more specialist compared to primary health care visits [10]. This may, in part, be due to the broader age categories used in the American study but could also reflect a real difference in the use of specialist and primary health care services in these two countries. The appropriate number of visits to primary health care and specialist services is difficult to establish but this research points to the continued need for a robust primary health care workforce.

Recent attention has focused on determining the relative impact of age and chronic disease burden on health system utilization [3]. In their 2011 report, the Canadian Institute for Health Information used data from the Canadian Survey of Experiences With Primary Health Care to report on the impact of chronic diseases [3]. In that study, the total number of chronic diseases was more important than age in predicting the annual number of health care visits made by seniors. In each of the age groups (65 to 74 years, 75 to 84 years, and 85 years and older), those with three or more reported chronic diseases had nearly three times the total number of primary health care visits compared to seniors with no reported chronic diseases. We report on the impact of age and disease count by looking at annual mean number of primary health care visits and find that, at a patient level, primary health care visits increased with both the number of diseases and also with age. The relative importance of these two factors (age and number of diseases) should be explored in future studies using statistical modeling.

This study has limitations common to all research using administrative data. These databases were constructed to serve a billing role and, thus, while they are rich in information, they were not created for research or disease ascertainment. To limit errors in our disease estimates we have used highly validated definitions of chronic diseases, but we acknowledge the possibility of over and under-ascertainment of disease. Furthermore, we estimated primary health care service use exclusively by looking at the number of visits and did not look at other health care services controlled by primary care, such as outpatient laboratory and diagnostic testing or number of prescriptions. Future research could explore these services to see if the patterns we identified hold.

## Conclusion

Primary health care plays a strong and vital role in managing chronic diseases. Results from this study show that the burden of care for persons in Ontario with chronic diseases, particularly the elderly, falls on the primary health care system. We found that, while people with multiple chronic diseases have more annual visits to their primary health care physician, the overall health system impact of multiple chronic diseases is still relatively small compared to the health care use of those with no or one chronic disease. These data support the continued investment in primary health care and the need for a strong focus on disease prevention to avoid the accumulation of chronic diseases.

## Abbreviations

SAS: Statistical analysis system.

## Competing interests

The authors declare that they have no competing interests.

## Authors’ contributions

EM contributed to the concept of the study and oversaw its implementation, helped guide the analysis, was the primary author and approved the final version of the manuscript. EG was responsible for the data analysis, participated in the editing of the manuscript and approved the final version of the manuscript. CB contributed to the implementation of the study, participated in the writing of the manuscript, and approved the final version of the manuscript. DM contributed to the concept of the study, oversaw its implementation, helped guide the analysis and participated in the writing and approved the final version of the manuscript. All authors read and approved the final manuscript.

## Authors’ information

EM is a new investigator at the C.T. Lamont Primary Health Care Research Centre and Bruyère Research Institute, clinician scientist at the Department of Family Medicine University of Ottawa, Canada and a research fellow at the Institute for Clinical Evaluative Sciences. EG was an analyst at the Institute for Clinical Evaluative Sciences at the time this research was completed. CB is a Research Coordinator at Institute for Clinical Evaluative Sciences. DM is a Senior Scientist, Ottawa Hospital Research Institute, Adjunct Scientist, Institute for Clinical Evaluative Sciences, Chair in Applied Public Health Sciences, CIHR/PHAC, Associate Professor, University of Ottawa and University of Toronto, and Associate Scientist, C.T. Lamont Primary Health Care Research Centre and Bruyère Research Institute.

## Pre-publication history

The pre-publication history for this paper can be accessed here:

http://www.biomedcentral.com/1472-6963/12/452/prepub
